# Study development and protocol for a cohort study examining the impact of baseline social cognition on response to treatment for people living with post-traumatic stress disorder

**DOI:** 10.1080/20008198.2022.2093036

**Published:** 2022-07-12

**Authors:** Chantelle Wiseman, Andrew D. Lawrence, Jonathan I. Bisson, James Hotham, Anke Karl, Stan Zammit

**Affiliations:** a Department of Population Health Sciences, Oakfield House, University of Bristol, Bristol, UK; b Cardiff University Brain Research Imaging Centre (CUBRIC), School of Psychology, Cardiff University, Cardiff, UK; c Division of Psychological Medicine and Clinical Neuroscience, Cardiff University, Cardiff, UK; d Avon and Wiltshire Partnership Trust, Bristol, UK; e Clinical Psychology and Affective Neuroscience, College of Life and Environmental Sciences (CLES), Psychology, Washington Singer Laboratories, University of Exeter, Exeter, UK

**Keywords:** PTSD, emotion recognition, social cognition, mentalisation, cohort study, psychological therapies, TEPT, reconocimiento de emociones, cognición social, mentalización, estudio de cohorte, terapias psicológicas, PTSD, 情绪识别, 社会认知, 心理化, 队列研究, 心理治疗

## Abstract

**Background:** Social cognitive impairments, specifically in facial emotion processing and mental state attribution, are common in post-traumatic stress disorder. However few studies so far have examined whether social cognitive ability impacts on PTSD recovery.

**Objective:** To examine whether baseline social cognitive abilities are associated with treatment outcomes following trauma-focused therapy for PTSD.

**Method:** This is a cohort study that will relate treatment outcomes post-discharge to baseline measures of social cognition (five tasks: Emotion Odd-One-Out Task (Oddity), Reading the Mind in the Eyes Task (RMET), Social Shapes Test (SST), Spontaneous Theory of Mind Protocol (STOMP), and Reflective Functioning Questionnaire (RFQ-8)) in people starting a course of psychological therapy for PTSD (target *N* = 60). The primary outcome will be pre- to post-treatment change in PTSD symptom severity (assessed using the PTSD Checklist for DSM-5). Secondary outcomes include functional impairment (assessed using the Work and Social Adjustment Scale), drop-out rate, and analyses differentiating participants with DSM-5 PTSD and ICD-11 PTSD and CPTSD. Regression models will be used to examine associations between baseline social cognitive performance and outcome measures while adjusting for potential confounders. Two pilot studies informed the development of our study protocol. The first involved qualitative analysis of interviews with nine participants with lived experience of mental health problems to inform our research questions and study protocol. The second involved trialling social cognitive tasks on 20 non-clinical participants to refine our test battery.

**Discussion:** This study will address a gap in the literature about whether abilities in social cognition in people living with PTSD are associated with treatment-related recovery.

**HIGHLIGHTS:**

- Impairments in social cognition are recognised in people with PTSD.
- Few studies have examined whether social cognitive ability is associated with recovery from PTSD.
- We present a study protocol, developed after pilot testing, to address this question.

## Abstract (es)

**Antecedentes:** Las deficiencias en la cognición social, específicamente en el procesamiento de las emociones faciales y de la atribución de estados mentales, son comunes en el trastorno de estrés postraumático (TEPT). Sin embargo, hasta el momento pocos estudios han evaluado si la habilidad cognitiva social tiene un impacto en la recuperación del TEPT.

**Objetivo:** Evaluar si las habilidades de cognición social de base están asociadas con los resultados del tratamiento después de la terapia centrada en el trauma para el TEPT.

**Métodos:** Este es un estudio de cohortes que relacionará los resultados posteriores al alta del tratamiento con las medidas de referencia de la cognición social mediante cinco pruebas: la tarea de la emoción no correspondiente (‘Emotion Odd-One-Out Task (Oddity)’), la tarea de lectura de la mente a través de la mirada (‘Reading the Mind in the Eyes Task (RMET)’), la prueba de las figuras sociales (‘Social Shapes Test (SST)’), el protocolo para la teoría de la mente espontánea (‘Spontaneous Theory of Mind Protocol (STOMP)’), y el cuestionario de funcionamiento reflexivo (‘Reflective Functioning Questionnaire (RFQ-8)’. Estas pruebas fueron realizadas en personas al iniciar el transcurso de la terapia psicológica para el TEPT (*N* objetivo = 60). El resultado principal será el cambio en la severidad de los síntomas del TEPT antes y después del tratamiento (evaluado utilizando la lista de verificación de síntomas de TEPT del DSM-5). Los resultados secundarios incluyen al deterioro funcional (evaluado mediante el cuestionario de trabajo y ajuste social, ‘Work and Social Adjustment Scale’ en inglés), la tasa de abandono, así como los análisis que diferencien a los participantes con TEPT según el DSM-5, y diferencien el TEPT y el TEPT complejo (TEPT-C) según la CIE-11. Se utilizarán modelos de regresión para examinar las asociaciones entre el rendimiento cognitivo social de referencia y las medidas de resultado mientras se ajustan por posibles variables de confusión. Dos estudios piloto sustentaron el desarrollo del protocolo del estudio. El primero involucró un análisis cualitativo de las entrevistas realizadas a nueve participantes con experiencias de problemas de salud mental para sustentar nuestras preguntas de investigación y el protocolo de estudio. El segundo involucró evaluar las pruebas de cognición social en veinte participantes sin condiciones clínicas para refinar la batería de pruebas.

**Discusión:** Este estudio busca estrechar la brecha en la literatura sobre si las habilidades en la cognición social en personas que viven con TEPT están asociadas con la recuperación vinculada al tratamiento.

## Abstract (zh)

**背景:** 社会认知障碍，特别是面部情绪处理和精神状态归因，在创伤后应激障碍中很常见。然而，迄今为止很少有研究考查社会认知能力是否会影响 PTSD 恢复。

**目的:** 考查基线社会认知能力是否与PTSD的聚焦创伤疗法后的治疗结果相关。

**方法:** 这是一项在开始接受一个PTSD 心理治疗疗程的人群（目标 *N* = 60）中进行的队列研究，将出院后治疗结果与社会认知的基线测量（5 个任务：情绪单外任务（Oddity）、眼中读心任务（RMET）、社会形态测试(SST)、自发心理理论 (STOMP) 和反射功能问卷 (RFQ-8)) 联系起来。主要结果将是治疗前后 PTSD 症状严重程度的改变（使用 DSM-5 PTSD 清单评估）。次要结果包括功能损伤（使用工作和社会调节量表评估）、流失率，以及区分DSM-5 PTSD 和 ICD-11 PTSD 和 CPTSD 参与者的分析。回归模型将用于考查基线社会认知表现与结果测量之间的关联，同时控制潜在混杂因素。两项试点研究为我们研究方案的制定提供了信息。第一项涉及对 9 名具有心理健康问题生活经验参与者的访谈定性分析，以为我们的研究问题和研究方案提供信息。第二个涉及对 20 名非临床参与者进行社会认知任务试验，以改进我们的测试组合。

**讨论:** 本研究将致力于解决文献中对于PTSD患者社会认知能力是否与治疗相关恢复有关的缺失。

## 1. Introduction

Post-traumatic stress disorder (PTSD) is a disabling mental health disorder that occurs in a minority of individuals exposed to a traumatic event, with a lifetime prevalence of approximately 7% in European adults (de Vries and Olff 2009). People with PTSD experience four key symptom clusters after exposure to severe stress: intrusion, persistent avoidance, hyperarousal, and trauma-related alterations in cognition and mood (APA, 2013). The International Classification of Diseases 11th edition also recognises a sister condition of complex PTSD (CPTSD), which has additional impairments in relationship difficulties, negative self-concept and affect dysregulation (WHO, 2018). Both disorders are associated with significant impairments in functional abilities (WHO, 2018).

The gold standard treatment for PTSD is a trauma-focussed psychotherapy such as trauma-focused cognitive behavioural therapy (TF-CBT) or eye movement desensitisation and reprocessing therapy (EMDR) (NICE, 2018). Cohort study data show that the natural course for PTSD is that only half will have recovered after 3 years (Perkonnig et al., 2005). Longer term follow-up studies show that between 7% and 29% of people still have significant symptoms of PTSD more than 15 years after their original trauma (Dai et al., 2016; Green et al., 1994; Morgan, Scourfield, Williams, Jasper, & Lewis, 2003). Trauma-related factors associated with recovery from PTSD include the type and intensity of the trauma, severity of injury, and loss of loved ones (Dai et al., 2016; Feng et al., 2007); non-trauma related factors include the level of social support and coping style (Dai et al., 2016). Evidence suggests that social support is an important protective factor against the development of PTSD and other psychopathologies (Yule, Houston, & Grych, 2019; Zalta et al., 2021).

Cognitive models aim to explain the development and maintenance of PTSD as the inability to recover from psychological trauma due to maladaptive processing of the traumatic memory. Fragmented and disorganised trauma memories are hypothesised to occur because of cognitive avoidance and emotion suppression. These core mechanisms are maintained by unhelpful negative self-attributions about the trauma, the self, and its sequelae (e.g. ‘The trauma happened because I am weak.’) (Ehlers & Clarke, 2000). Evidence-based trauma-focussed talking therapies address these underlying maladaptive cognitions and support trauma memory integration into a coherent narrative that includes making meaning of the traumatic experience (Brown, Belli, Asnaani, & Foa, 2019a). Studies report that a considerable proportion of patients do not benefit from therapeutic approaches because they are emotionally demanding (Cloitre 2015; Lewis, Roberts, Andrew, Starling, & Bisson, 2020) and there is an 18% dropout rate (Imel, Laska, Jakupcak, & Simpson, 2013). Identifying factors that influence recovery from PTSD could inform prediction models and individualised therapy, as well as the development of new therapeutic targets to improve treatment outcomes.

One possible impediment to treatment recovery, particularly amongst those who present with CPTSD, is impairment in social cognition. Social cognition involves processing information about the self, others, and the social world (Beer & Oschner, 2006). Individual differences in social cognition may be a key determinant of whether the behaviour of others is perceived as supportive (Costa-Cordella, Arevalo-Romero, Parada, & Rossi, 2021).

Social cognition is a multi-dimensional construct (Happé, Cook, & Bird, 2017; Pinkham et al., 2014; Schaafsma, Pfaff, Spunt, & Adolphs, 2015). While there is no definitive ontology of social cognitive processes, studies in clinical populations have converged on a replicable three-factor structure (Mancuso, Horan, Kern, & Green, 2011; Riedel, Horan, Lee, Hellemann, & Green, 2021) – ‘low-level’ (emotion processing, social perception), ‘high-level’ (mentalisation) and ‘attributional bias or response style’. Emotion processing and perception is the ability to attribute emotions to static and dynamic configurations of facial movements. Social perception refers to decoding and interpreting social cues in others. Mentalisation or Theory of Mind (ToM) is the ability to attribute mental states to others and oneself. Attributional style or bias is the tendency to attribute the cause of events to the self, others or the environment.

Broad social cognitive deficits and differences have been identified in people with PTSD, including problems correctly attributing emotions and inferring the mental states of others and the self (Couette, Mouchabac, Bourla, Nuss, & Ferreri, 2020; Stevens & Jovanovic, 2019). A recent meta-analysis of these underlying domains of social cognition, which included 19 studies involving 565 individuals with PTSD and 641 controls, found that individuals with PTSD scored lower on overall social cognitive functioning, particularly in mentalisation, with a medium effect size for overall social cognition impairment in the PTSD group (Janssen et al., 2022).

It has been hypothesised that people with poor or atypical social cognition may have a lower threshold for developing PTSD after exposure to a trauma (Sharp, Fonagy, & Allen, 2012). Attachment style has been hypothesised to be a key factor in the relationship between social cognition and PTSD, with social cognitive abilities mediating the relationship between childhood attachment and PTSD risk and resilience (Sharp et al., 2012).

Social cognition may also be important for an individual's ability to benefit from psychological therapy. A closely related construct, psychological mindedness, has been described as a key factor in understanding why some people benefit more from psychological therapies than others. Psychological mindedness is ‘A person's ability to see relationships among thoughts, feelings and actions, with the goal of learning the meanings and causes of his experiences and behaviours’ (Appelbaum, 1973). Individual differences in social cognition could explain why some individuals fare less well in their recovery from trauma and response to psychological therapies (Sharp et al., 2012). Difficulties with social cognition, for example with identifying the facial cues and intentions of the therapist, could lead to a lack of perceived support, poorer therapeutic relationship and adversely impact the outcome of therapy (Costa-Cordella et al., 2021; Couture et al., 2006). Mental state attribution errors have been associated with poorer treatment response in alcohol dependence disorders (Rupp, Derntl, Osthaus, Kemmler, & Fleischhacker, 2017), psychosis (Jung, Wiesjahn, & Lincoln, 2014) and borderline personality disorder (Kvarstein et al., 2020).

To date, there is only very limited evidence that socio-emotional cognition is associated with PTSD recovery. Venta, Hatkevich, Mellick, Vanwoerden, and Sharp (2017) found that baseline social-cognitive ability, measured using the movie for the assessment of social cognition (MASC; Dziobek et al., 2006) predicted improvement in PTSD symptoms following combined cognitive–behavioural/family systems therapy in a group of in-patient adolescents. Using functional MRI, Farrow et al. (2005) found that, following psychotherapy for PTSD, mentalisation-related brain activation increased in several areas of the default network (including posterior cingulate, medial prefrontal cortex and superior temporal gyrus).

To help address this knowledge gap we will examine whether social cognitive abilities in people with PTSD are associated with the degree of recovery following a trauma-focused psychological therapy. We hypothesise that lower baseline social cognitive abilities will be associated with impaired recovery from PTSD following therapy. Here we describe the protocol for this study, and the two pilot studies that informed its development.

## 2. Protocol development

To develop our protocol, we undertook two preliminary studies. The first involved a qualitative analysis of interviews and focus groups with people who have lived experience of a mental health disorder. The second tested the acceptability of the planned social cognitive task battery. We briefly discuss these studies here.

### 2.1. Pilot Study 1: qualitative study of participants recruited from patient and public involvement groups

This pilot aimed to explore patient views on our study topic and study design. We recruited nine participants (four with PTSD/CPTSD, four with affective disorders, one with emotionally unstable personality disorder (EUPD)) from established PPI (Patient and Public Involvement) groups based at the universities of Cardiff, Exeter and Bristol. Convenience sampling was used by a researcher contacting the groups and inviting members into this study. Between March and August of 2019, we conducted two focus groups (one with three participants and one with four) and separately interviewed two individuals (one with CPTSD and one with EUPD). Both the focus groups and interviews were semi-structured using the same topic list, which explored participants’ understanding of social cognition, trauma, and PTSD, as well as asking for feedback on our initial protocol design. Interviews were transcribed and thematic analysis was used (Braun & Clarke, 2008) with double-handed coding to analyse the data. We completed member-checking of the findings with four participants. £20 renumeration in gift vouchers, as well as travel expenses, were provided for participants.

**Figure 1.**
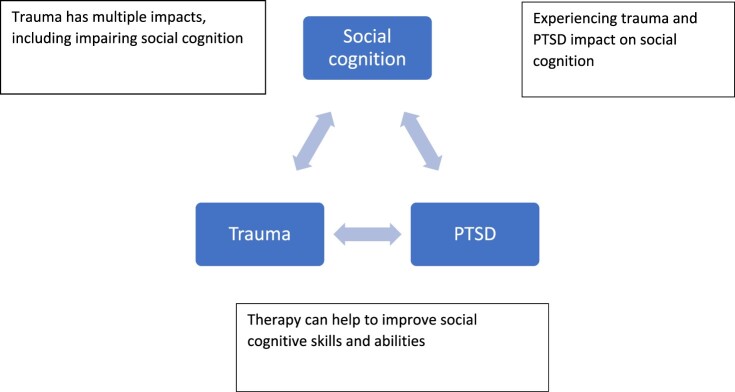
A summary of the interplay between social cognition, trauma and PTSD identified in Pilot Study 1.

We identified three themes: (1) Social cognition is variable within an individual across time and context. (2) Impaired social cognitive ability could increase the risk of PTSD. (3) Trauma exposure and PTSD can affect social cognition. More detailed information is supplied in the Appendix and is visualised in Figure 1.

Table 1 shows the feedback on our initial study protocol and how this led to further development of the protocol. Key feedback was that testing should be more focussed to minimise potential distress and so we developed our protocol accordingly.

**Table 1.** PPI findings relevant to our study protocol from Pilot Study 1.

Feedback from participants regarding this project	Detail	How we modified our main protocol
Length of testing time	Participants thought people with PTSD could manage 2 hours of testing with breaks	Tasks restricted to 1–2 hours; testing will be flexible to allow for breaks
Conducting testing	Clinical staff seen as advantageous as would understand if participants become distressed Having a friend or a carer attending with the participant would be of benefit.	Testing will be conducted by clinical staff A companion will be allowed to accompany the participant for support, but will not be allowed to confer for the tasks
Research tasks	Recognition that people living with PTSD can have impaired concentration and motivation. Tasks should be focussed and relevant	Tasks will be focussed to minimise participant burden
Some tasks can cause distress	Social cognition tasks often involve emotion processing. However, one participant reported looking at faces for a prolonged period upsetting and caused nightmares. Detailed questions on childhood trauma could provoke distress; emotions may not be suitably contained in a research setting	We will be aware of this as a potential issue with data collection, but will retain emotion processing tasks as these provide relevant data Trauma measures should be focussed and not excessively intrusive
Accessing participants clinical notes	Participants felt that relevant (i.e. mental health) notes could be accessed for study purposes with permission	We will access mental health notes for information on the trauma, with informed consent.

### 2.2. Pilot Study 2: feasibility study in a non-clinical sample

We recruited 20 non-clinical participants from the University of Bristol and piloted a battery of pre-existing social cognitive tasks during August 2019. The aim of this study was to ensure that the tasks and duration of testing were acceptable, while selecting several types of measures that were likely to produce individual differences, rather than testing a specific a priori model of social cognition. The measures used were: The Emotion Odd-One-Out Task (Oddity), The Reading the Mind in the Eyes Task (RMET), The Social Shapes Test (SST), Spontaneous Theory of Mind Task (STOMP), and the Reflective Functioning Questionnaire (RFQ-8). They are described in greater detail below. We also included a verbal IQ measure (Spot the Word Test (STW)), a measure of attachment style (The Adult Attachment Questionnaire (AAQ)) and a feedback questionnaire. Participants received a £10 gift voucher.

**Figure 2.**
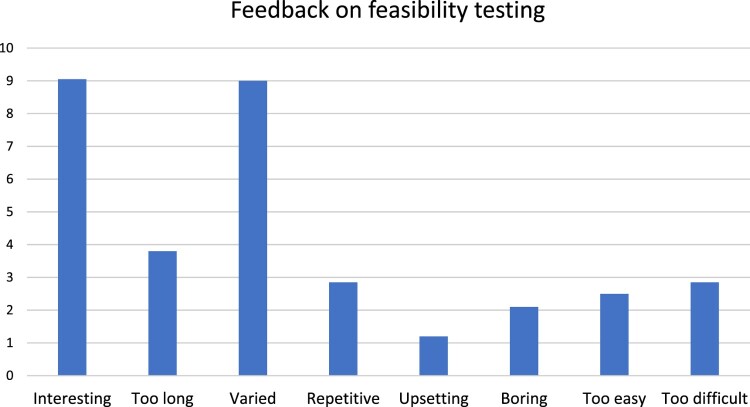
Feedback on battery of tasks from Pilot Study 2.

The battery of tasks took participants between 40 minutes to 1 hour to complete. Feedback was positive, and participants found the tasks interesting and varied (mean scores 9/10); see Figure 2 for more information.

## 3. Protocol

### 3.1. Ethical approval

Ethical approval was granted by the Health Research Authority and the Oxford B Regional Ethics Committee in June 2019.

### 3.2. Study design

This is a cohort study of patients with DSM-5 PTSD and ICD-11 PTSD or Complex PTSD who will be assessed before and after a course of psychological therapy.

### 3.3. Recruitment and inclusion criteria

Participants will be recruited from two specialist PTSD treatment services in the South-West of England and South Wales. Patients with a clinical diagnosis of either DSM-5 PTSD or ICD-11 PTSD or CPTSD who are at the top of the waiting list for a trauma-focused therapy (either EMDR or TF-CBT) will be contacted by letter or telephone and invited into the study. Participants will undergo baseline data collection as close to commencement of therapy as possible, and no later than four weeks after their initial appointment. All patients complete the PCL-5 and ITQ at the time of referral and at their initial therapy appointment. Exclusion criteria are age <18 years, lack of English fluency, and a primary diagnosis other than PTSD.

### 3.4. Sample size calculation

An a priori power calculation using the ‘power’ command in Stata v16 (STATA, 2019) indicated that 50 participants are needed to have 80% power to detect a moderate effect size of *d *= 0.4 for a standardised score change in PCL-5 per SD change in social cognition score. We, therefore, aim to recruit 60 individuals to allow for 20% dropout (Imel et al., 2013).

### 3.5. Procedure: baseline assessment

**Figure 3.**
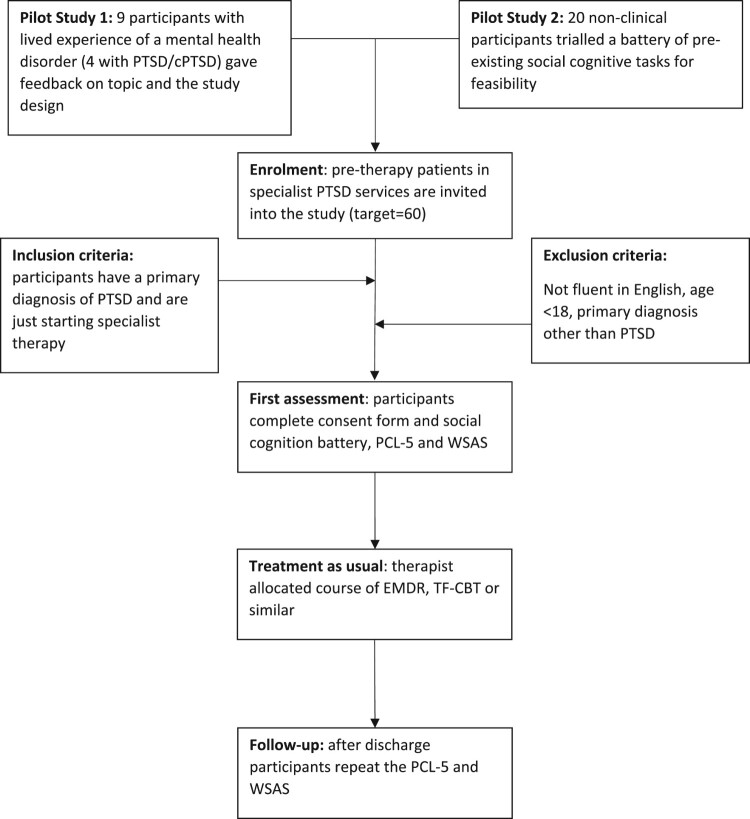
Flow chart of the study design.

Figure 3 details the pathway for participants in the study. The initial plan was for participants to meet with a member of the research team and complete the study on a laptop with the researcher there for guidance and support. However, due to the COVID-19 pandemic, this was amended to run remotely on an online platform, with the consent process completed via telephone and through an interactive consent form (Lourenco & Tasimi, 2020).

The participants are sent a link to the testing website Qualtrics (Qualtrics, Provo, UT, 2021) that they can access at home on their own electronic device. They are advised to use a laptop or tablet device so the images are clearer, but the study has also been adapted to work on a smartphone if that is all that is available. Telephone support is available from one of the research team throughout the testing appointment. The session is bookended by a researcher contacting the participant after two hours to provide a debrief. The measures used are described below. Because the tasks are completed at home there is no direct monitoring of participants or time limit on tasks.

### 3.6. Social cognitive tasks

We use five social cognitive tasks, selected to broadly cover the domain of social cognition with varying levels of naturalism and be sensitive to individual differences, rather than testing a specific a priori model of social cognition (Fan, Dal Monte, & Chang, 2021; Lakhani, Bhola, & Mehta, 2021; Schaafsma et al., 2015). The first two, The Emotion-Odd-One-Out (Oddity) Task and The Reading the Mind in the Eyes Task (RMET), examine static face emotion processing. The Social Shapes Test (SST) and The Spontaneous Theory of Mind Task (STOMP) measure attribution of mental states to animate objects and movie characters, respectively. The Reflective Functioning Questionnaire (RFQ) is a self-reported assessment of mentalising about the self and others.

#### 3.6.1. Facial emotion processing

##### 3.6.1.1. Emotion-Odd-One-Out (‘Oddity’) task

We use the Face Emotion subset of a Multi-Condition Odd-One-Out (‘Oddity’) judgement task. This is a perceptual odd-one-out discrimination task where participants must identify which of three faces is displaying a different ‘basic’ emotion (Ekman, 1992) from the other two. The task consists of 36 trials, each with three images of different identity faces presented in a triad (bottom left, top middle and bottom right). The faces are generated in FaceGen Modeller 3.5 (Singular Inversions, 2022) and are designed to make the faces gender and ethnicity neutral. They are shown in greyscale and are hairless. The trials alternate between the images being face-on or rotated 45° to the left or right. The odd-one-out is counterbalanced for location (appearing in the left, middle and right positions an equal number of times). This task is based on the work of Palermo, O’Connor, Davis, Irons, and McKone (2013) and has been validated against other measures of face and emotion processing, including the RMET (Coad et al., 2020).

##### 3.6.1.2. Reading the Mind in the Eyes (RMET)

Originally designed as a test of mentalising, the RMET is now considered an explicit emotion labelling task (Kittel, Olderbak, & Wilhelm, 2021; Oakley, Brewer, Bird, & Catmur, 2016) wherein participants select one of four complex emotional states to best describe a greyscale photograph, cropped to depict the eye region of a series of adult faces (Baron-Cohen, Wheelwright, Hill, Raste, & Plumb, 2001). There are 36 sets of pictures. Originally designed to assist in the detection of autism, this task has been used in other clinical populations and as a measure of individual differences in complex emotion recognition in healthy adults (Baron-Cohen et al., 2001). Although subject to various criticisms (Kittel et al., 2021), it has been used in a wide variety of studies, often as a measure of nomological validity for newer social cognition measures.

#### 3.6.2. Mentalising

##### 3.6.2.1. The Social Shapes Test (SST)

ToM is so automatic and pervasive that humans even attribute complex mental states to animated abstract shapes, although the extent of such attributions can differ widely across individuals (Tavares, Barnard, & Lawrence, 2011). In the SST participants are shown 23 short video clips of coloured cartoon shapes interacting (Brown et al., 2019b). After each video, they must pick the statement that most accurately describes it from a choice of four. This test is thought to minimise linguistic and cultural biases (Brown et al., 2019b). This task has recently been used in online studies with patients with autism (Brown, Chabris, & Heck, 2021) and has good convergent validity with other measures of ToM (Brown et al., 2019b).

##### 3.6.2.2. The Spontaneous Theory of Mind Protocol (STOMP) task

A mental state attribution task measuring participants’ spontaneous descriptions of the beliefs, emotions, and goals of characters in naturalistic short movies. Participants watch a silent 90-second movie clip. We use a scene from a teenage comedy in which several teenage girls are trying to take revenge on a boy who dated them all. Immediately post-viewing, participants are instructed to describe the scene and encouraged to write around 7–10 lines of text. The response is then coded for the use of mental state vs. non-mental state language. Tasks of this nature are useful as participants are unlikely to score full marks due to a ceiling effect, a potential issue with some other social cognition tasks (Dodell-Feder, Lincoln, Coulson, & Hooker, 2013). This task has good reliability and correlates well with other higher-order story-based ToM tasks, but less so with RMET (Rice & Redkay, 2015).

#### 3.6.3. Self-reported assessment of mentalisation

##### 3.6.3.1. Reflective Functioning Questionnaire 8 items (RFQ-8)

A questionnaire consisting of eight self-report statements measuring perceived internally-focussed mentalisation abilities (Fonagy et al., 2016). The items report the participants’ self-reported tendencies to reflect on/understand their own and others’ thoughts and feelings. From the eight statements a score is derived for hypomentalising (struggling to understand the mental states of oneself and others), uncertain reflective functioning (a concrete, rigid way of mentalising), and hypermentalising (attributing intent to the behaviour of others that may not actually exist). This measure has been tested in clinical populations of patients with eating disorders and personality disorders and has been shown to adequately differentiate clinical and non-clinical groups (Fonagy et al., 2016). Further, baseline RF has been shown to predict psychotherapy outcomes in patients with depression (Ekeblad, Falkenström, & Holmqvist, 2016).

### 3.7. Additional measures

These include the PCL-5 and WSAS (described under ‘Outcome Measures’), as well as measures of verbal IQ, attachment style, personality dysfunction and trauma history as potential confounders to adjust for in our analyses.

#### 3.7.1. Spot the Word task

Verbal ability and education are correlated with social cognitive performance (Brown et al., 2019b; Kittel et al., 2021) and IQ is associated with PTSD (Breslau, Lucia, & Alvarado, 2006). In this task, 60 pairs of items comprised of one word and one non-word are presented, and participants must choose the word (Baddeley, Emslie, & Nimmo-Smith, 1993). This task has adequate validity and reliability when compared with other verbal IQ tasks such as the National Adult Reading Test (Baddeley et al., 1993).

#### 3.7.2. The Adult Attachment Questionnaire

Attachment style is associated both with social cognition and PTSD (Sharp et al., 2012; Venta et al., 2017). In this well-validated measure, participants review 17 statements describing their adult relationship style using a Likert-type scale (Simpson, Rholes, & Philips, 1996). This provides scores on Avoidant and Ambivalent attachment, and those with secure attachment patterns should obtain low scores on both.

#### 3.7.3. Standardised Assessment of Personality – Abbreviated Scale (SAPAS)

Personality disorders such as borderline personality disorder are associated both with deficits in social cognitive ability (Roepke, Vater, Preissler, Heekeren, & Dziobek, 2013) and impairments in recovery from co-occurring mental disorders (Newton-Howes et al., 2014). We have included a brief, validated measure of personality for this potential confounder (Moran, Leese, Tennyson, & Walters, 2003). An 8-item questionnaire that requires yes/no answers from participants, it is a valid and reliable screening questionnaire for personality disorder (Moran et al., 2003).

#### 3.7.4. Life Events Checklist for DSM-5 (LEC-5)

A self-report checklist of 16 potentially traumatic events to have occurred throughout the lifetime (Weathers et al., 2013a). We use a modified version that includes two additional measures of childhood trauma, sexual and physical abuse. This provides us with information on the number of traumatic events participants have experienced, witnessed, or learned about in their lives. Earlier versions of this scale had sufficient convergent validity with other scales measuring trauma exposure (Weathers et al., 2013a)

### 3.8. Outcome measures

Our primary outcome is change in PTSD symptom severity pre- to post-therapy (assessed using the PCL-5). Secondary outcomes are change in functional ability pre- to post-therapy (assessed using the WSAS), and treatment drop-out, defined as therapy ending without therapist agreement.

#### 3.8.1. PTSD checklist for DSM-5 (PCL-5)

This symptom checklist enquires about the four clusters of PTSD symptoms according to the DSM-5 (reliving, avoidance, hyperarousal, and cognitive beliefs) that have occurred over the previous month (Weathers et al., 2013b). They are measured on a Likert-type scale from 0 (not at all) to 4 (extremely). These can be summed to provide a total score (range 0–80). This measure has been shown to fit with DSM 5's four factor model for PTSD, as well as having strong reliability and validity (Blevins, Weathers, Davis, Witte, & Domino, 2015). The PCL-5 is routinely used as a progress measure in the services that we are recruiting from, which will allow us to supplement data if participants fail to engage with our follow-up.

#### 3.8.2. Work and Social Adjustment Scale (WSAS)

Our secondary outcome is improvement in daily functioning following psychological therapy for PTSD. The WSAS is a five-item questionnaire on a Likert-style scale that measures how severely a psychiatric condition impacts on daily functioning, such as the ability to work and to socialise (Mundt, Marks, Shear, & Greist, 2002). This scale has correlated well with other measures of functioning in a trauma-exposed sample (Hussain, Weisaeth, & Heir, 2011).

### 3.9. Complete test-battery at time 1

The final battery consists of five social cognition measures, as well as the outcome measures and information on confounders. It takes approximately 100 minutes to complete. At the end of the battery is a brief feedback form consisting of open and closed questions that we can use to ensure the study is not causing undue distress to participants. Table 2 summarises the tasks in order of presentation to the participant. Trauma and PTSD measures are placed at the end of the battery to prevent any distress caused by these questionnaires impacting on responses to other parts of the study.

**Table 2.** Task battery for Time 1 Assessment in order; social cognition tasks shown in bold.

Task name	Description
**The Social Shapes Test** (Brown et al., 2019a)	Participants are shown 23 short video clips of coloured cartoon shapes interacting. After each video they select from a choice of four statements which best describes the scene.
**The Emotion Odd-One-Out Task** (based on Coad et al., 2020)	A set of three different faces is shown; two pictures show actors depicting the same basic emotion, one shows a different basic emotion. There are 36 sets. The participant must select the odd one out.
**The Reflective Functioning Questionnaire** (8 items) (Fonagy et al., 2016)	A questionnaire consisting of eight self-report statements measuring self-assessed mentalisation tendencies.
**Reading the Mind in the Eyes** (Baron-Cohen et al., 2001)	Participants select one of four complex emotional states to describe an image of a pair of eyes. There are 36 sets of pictures.
Standardised Assessment of Personality – Abbreviated Scale (Moran et al., 2003)	An 8-item questionnaire with yes/no answers related to problematic interpersonal functioning.
**Spontaneous Theory of Mind Protocol** (Rice & Redkay, 2015)	Participants watch a 90-second movie clip without the soundtrack. They are then instructed to ‘Please describe this scene’ and encouraged to write around 7–10 lines of text.
Adult Attachment Questionnaire (Simpson et al., 1996)	Participants review 17 statements describing their adult relationship style using a Likert-style scale.
Spot the Word (Baddeley et al., 1993)	Sixty dyads consisting of one real word and one non-word are presented, and participants have to identify the real word.
Life Events Checklist for DSM-5 (LEC-5) (Weathers et al., 2003a)	Details exposure to 18 specific traumas and one ‘other’ trauma.
PTSD Checklist for DSM-5 (PCL-5) (Weathers et al., 2003b)	Lists PTSD symptoms and participants rate how severely they have experienced these in the past month; 20 items.
Work and Social Adjustment Scale (WSAS) (Mundt et al., 2002)	Participants rate using a Likert scale how severely their daily functioning is affected by PTSD. Five items.
Feedback	Participants provide feedback through open and closed questions on the tasks.

### 3.10. Additional information from the participants’ clinical notes

In addition, we request informed consent from participants to access their electronic mental health notes. This allows us to obtain important additional study information whilst reducing participant burden. We complete the DSM-5 Criterion A questionnaire from the clinical notes to obtain information on the type and severity of trauma exposure that precipitated the participant developing PTSD (Weathers et al., 2013b). We also collect data on the ITQ, described below, from the first and last therapy appointments, and other relevant information including other mental health diagnoses at any point, any medications, physical health conditions, social support, and drop-out from therapy. Previous and other current mental health diagnoses are possible confounders as autism and schizophrenia are particularly associated with social cognitive ability (Fernandes, Cajao, Lopes, Jeronimo, & Barahona-Correa, 2018).

#### 3.10.1. The International Trauma Questionnaire (ITQ)

The ITQ is a validated, self-report 18-item Likert questionnaire for assessing symptoms and diagnoses of ICD-11 PTSD and CPTSD (Cloitre et al., 2018). We use it to allow us to distinguish between ICD-11 PTSD or CPTSD for our secondary analyses. It has been used to detect and differentiate PTSD and CPTSD in both clinical and community samples (Cloitre et al., 2018).

### 3.11. Procedure: follow-up session at time 2

After the initial testing session, the patients continue their treatment as usual. They are then contacted again after they have been discharged to repeat the outcome measures (PCL-5 and WSAS) online. Participants receive a £20 gift voucher for participation.

### 3.12. Data management

Participants are assigned random study identification numbers, which are used on the study platform Qualtrics (Qualtrics, Provo, UT). Personal information from consent forms is securely stored separately according to the University of Bristol's secure data policy.

### 3.13. Data analysis

Data analysis will be completed using STATA statistical software (STATA, 2019). SST, RMET and the Oddity task are all multiple-choice tasks, and accuracy of performance will be scored according to published criteria. The RFQ-8 has a specific scoring system to follow that produces certain and uncertain reflective functioning scores (Fonagy et al., 2016). We will score the STOMP by comparing the relative amount of mental state versus non-mental state language used in the free text (Orr & Gilead, 2021; Rice & Redkay, 2015). We will examine the inter-correlation between social cognition task measures (Warnell & Redcay, 2019) and if appropriate apply data reduction techniques (e.g. principal components analysis, PCA), which may help to reduce the number of statistical tests (Budaev, 2010). Otherwise, we will analyse the results for each task separately.

The primary outcome measure will be treatment response measured as the change in PCL-5 score pre- to post-therapy. As secondary outcomes, we will also examine change in DSM-5 PTSD symptom-cluster scores, change in the proportion meeting criteria for DSM-5 PTSD, ICD-11 PTSD, and ICD-11 CPTSD post-therapy, drop-out from therapy and change in functional ability measured as change in WSAS scores pre- and post-therapy. The impact of confounding in our analyses will be examined by comparing unadjusted models to models adjusted for age, gender, personality dysfunction, other psychiatric conditions, attachment style, verbal IQ, severity of trauma history and type of PTSD (ICD-11 PTSD versus CPTSD). As an exploratory analysis, we will also examine results separately in those with ICD-11 PTSD and CPTSD, although statistical power comparing these subgroups will be limited, and evidence suggests outcomes might be similar in these groups (Hoeboer et al., 2021). Regression models will be used to estimate effect sizes and 95% confidence intervals for our outcomes per unit-difference in social cognition measures. We will also derive estimates of prediction including sensitivity, specificity, positive predictive values, and area under the curve to determine whether specific thresholds of social cognitive scores have potential clinical value in predicting response to therapy.

## 4. Discussion

We have designed a cohort study to assess whether social cognition in people with PTSD is associated with recovery following trauma-focused therapy. We hypothesise that poorer social cognitive ability at baseline will predict poorer treatment outcomes. Discussions with individuals with lived experience and feasibility testing in a non-clinical population have influenced our study design. As we collect data both on a DSM 5 diagnosis of PTSD (via the PCL-5) and for an ICD-11 diagnosis of PTSD or CPTSD (using the ITQ) we hope to be able to assess the impact of baseline social cognition on treatment outcome in these three different diagnostic groups.

We have developed a testing battery to measure social cognition that includes previously validated tasks that map to several aspects of social cognition, including basic and complex face emotion processing and mental state attribution (aka ToM or mentalising). We use both forced-choice and open-ended response tasks, as well as a self-report measure. The measures were selected to broadly cover the domain of social cognition with varying levels of naturalism and be sensitive to individual differences, rather than testing a specific a priori model of social cognition (Schaafsma et al., 2015). We have also included measures of potential confounders to allow us to determine the extent to which any association found between social cognition and response to therapy is likely to be causal. We use the PCL-5 for our primary outcome as this is a well-validated and frequently used research tool (Blevins et al., 2015) that is routinely used in the services we are recruiting from.

We acknowledge there will be challenges with our data collection and study design. Defining and measuring social cognition is difficult, given the lack of a formal ontology of social cognition (Happé et al., 2017; Poldrack et al., 2011; Schaafsma et al., 2015). For example, a simple emotion processing task using pictures of faces can be considered to measure emotion perception, semantic emotion knowledge, affective ToM, or cognitive empathy, depending on the perspective of the researcher. Social cognition tasks are also prone to being influenced by socio-economic status, educational attainment and verbal IQ (Brown et al., 2019b). For this reason, we have included both verbal and non-verbal measures of social cognition and a verbal IQ measure. Finding tasks that are appropriate for a clinical population also requires consideration. Three of the tasks we use (RMET, RFQ-8 and SST (or variants of it)) have been validated in clinical populations (Baron-Cohen et al., 2001; Brown et al., 2021; Fonagy et al., 2016). The emotion odd-one-out task is based on similar face identity odd-one-out tasks that have been used in clinical populations (Behrmann, Lee, Geskin, Graham, & Barense, 2016). As far as we are aware The STOMP task has only been used in non-clinical populations. However, we have included it as a more naturalistic (movie-based) measure, sensitive to individual differences in social cognitive abilities, which shows good convergent validity with other narrative-based measures of ToM and cognitive empathy (Rice & Redcay, 2015; Warnell, De La Cerda, & Frost, 2021).

One issue we foresee is the potential high degree of correlation among the different social cognition measures (Schaafsma et al., 2015) and the consequent impact on statistical power when conducting multiple statistical tests. SST scores correlate significantly with RMET scores (Brown et al., 2019b), RMET and emotion odd-one out tasks have also demonstrated a significant correlation (Coad et al., 2020; Palermo et al., 2013). This indicates there is some degree of commonality in what is being measured with these tasks. However, not all studies have shown evidence of correlation between different social cognition tasks (e.g. STOMP and RMET scores do not appear to correlate) (Rice & Redkay, 2015; Warnell & Redcay, 2019), and seemingly similar concepts such as mentalisation and mind-mindedness (recognising that social contacts have their own independent agency) can be independent (Pequet & Warnell, 2020) (the so-called ‘jingle-fallacy’) (Dang, King, & Inzlicht, 2020). In addition, the RFQ-8 only weakly correlates with RMET (Fonagy et al., 2016), perhaps unsurprisingly given the typically low correlation between self-report and behavioural measures (Dang et al., 2020). We plan to address the issue of the potential high correlation between different social cognition measures by using dimension reduction techniques (e.g. PCA) which may help when testing hypotheses to reduce the number of statistical tests (Budaev, 2010).

As far as we are aware, this is the first study to examine the association between a comprehensive battery of social cognitive measures and response to trauma-focused therapy in an adult sample. The results of this study may help us to better understand why patients vary in their response to evidence-based therapies for PTSD.

## Ethics approval

Ethical approval was granted by the Health Research Authority and the Oxford B Regional Ethics Committee in June 2019 for Pilot Study 2 and for the main study, IRAS number 263222. Pilot Study 1 was discussed with the Health Research Authority and University of Bristol Ethics Committee and deemed to be Patient and Public Involvement in Healthcare, and therefore did not require ethical approval. Informed consent and consent to publish research findings, including quotations, was obtained and copies of relevant correspondence are available on request.

## Data Availability

The data that support the findings of the preliminary studies are available on request from the corresponding author, CW. The data are not publicly available due to the small sample size meaning information is present that could compromise the privacy of research participants.

## Appendix. Themes from qualitative interviews with PPI group members

1. Social cognition is variable within an individual across time and context

Participants did not conceive of social cognitive ability as something that is fixed and immutable, but rather as one that can vary both over longer time scales (e.g. lifespan development, adaptation) and also over shorter time scales as a function of their current situation. They described improvements with age and psychological therapy, and deterioration when they had a lack of social support and also as a short and longer-term consequence of trauma.
Participant 5: It's also interesting because I think that my social cognition has improved with age … And my understanding of my mental health. Before I never really understood what was going on … is it anger, is it this, that? … But now I’m able to make that understanding of it.
Participant 4: So, I felt like that CBT taught me how to be more aware of those emotions because I wasn't very good at the start and I’m not saying I’m great now, but I’m much better than I was. Participants were divided as to whether someone with poor social cognition could benefit from psychological therapy; some thought that not having these skills to draw on would make the process of therapy too difficult.
Participant 5: If you can't understand yourself or understand others, I don't think talking therapies would be very … I think that would be hard. Other participants thought that with a therapist that is experienced and patient, people with impaired social cognition could improve these abilities.

2. Reduced social cognitive ability could increase the risk of PTSD

Participants discussed being able to understand that deficits in social cognition, i.e. understanding the motivations and internal processes of themselves and others, might predispose to the development of PTSD.

Participant 4: So, I’m okay at understanding people's emotions in a room like this, but I don't think I’m very good at assessing my emotions because this thing [PTSD] crept up on me and I didn't realise. Some participants also described that around the time of the traumatic event, they focussed on caring for others, such as elderly relatives, children and junior colleagues.
Participant 3: After the incident my first feelings were to protect them and to protect my daughter, I didn't think of myself. I was so scared, obviously. They considered this to also be contributory to their PTSD development because they neglected their own well-being in a time of extreme stress to look after others.
Participant 4: I was the only one who could deal in that particular situation and then I was the only one afterwards who couldn't deal with that particular situation in my own emotions.
Participant 3: It's weird because that is actually a strength, we’ve all shown great strength!
Participant 4: Yeah.
Participant 3: But it didn't do us any good in the end.
Participant 4: No, we paid a price.

3. Trauma exposure and PTSD can affect social cognition

Some of the participants spoke of the long-term impacts of trauma exposure on their mental functioning.

Participant 8: Trauma! … It just numbs your brain, parts of your brain just shut down … I just couldn't think beyond a certain level, couldn't write it down, couldn't work with it … . Especially around maths. I couldn't think … It's like the brain wouldn't function. Specifically, they discussed the impact on their emotional stability, avoidance, and ability to relate to others.
Participant 5: I’m thinking that the trauma I had from my childhood … was so traumatic, that I never dealt with that for 40 years … I actually was very unfeeling towards other people and very unfeeling towards myself, I never went there, so it affected my ability to deal with my own emotions, I just shut them off.
Participant 9: Those people have just got blunted. I don't think they necessarily started life that way but whatever has traumatised them has meant that they are, there are signals they don't read. Anger and feeling unsafe with others were key to this theme.
Participant 8: I could become angry … verbally challenging is the word … Because the fear, anger, reaction was too overwhelming, and I wasn't doing myself any good. In addition, some participants recognised that experiencing PTSD meant their social cognition was acutely impacted. It was unclear in some cases whether the participants thought the impact on their social cognition was a consequence of the trauma or part of the symptomatology of PTSD. Other participants were unclear whether social cognition was affected by the trauma and PTSD, or whether poor social cognition preceded the trauma.
Participant 4: That's the thing though … does one affect the other, or … which way round is it? For some participants the directionality did not matter; they expressed that PTSD is such a complicated disorder the exact causative mechanism was not important and was unlikely to be the same for all patients.

The conceptualisations of the relationship between trauma, social cognition and PTSD highlighted from discussions with these participants is shown in Figure 1.
